# Colony environment and absence of brood enhance tolerance to a neonicotinoid in winter honey bee workers, *Apis mellifera*

**DOI:** 10.1007/s10646-024-02758-8

**Published:** 2024-05-23

**Authors:** Manon Bovier, Domenic W. Camenzind, Andrew F. Brown, Lukas Jeker, Gina Retschnig, Peter Neumann, Lars Straub

**Affiliations:** 1 Institute of Bee Health, Vetsuisse Faculty, University of Bern, Bern, Switzerland; 2 University of Freiburg, Freiburg, Switzerland; 3 Swiss Bee Research Centre, Agroscope, Bern, Switzerland; 4 Faculty of Science, Energy and Environment, King Mongkut’s University of Technology North Bangkok, Rayong Campus, Rayong, Thailand; 5 Centre for Ecology, Evolution, and Behaviour, Department of Biological Sciences, Royal Holloway University of London, Egham, UK

**Keywords:** Agriculture, Behaviour, Ecotoxicology, Eusociality, Insecticide, Survival

## Abstract

In eusocial insects, worker longevity is essential to ensure colony survival in brood-free periods. Trade-offs between longevity and other traits may render long-living workers in brood-free periods more susceptible to pesticides compared to short-lived ones. Further, colony environment (e.g., adequate nutrition) may enable workers to better cope with pesticides, yet data comparing long vs. short-living workers and the role of the colony environment for pesticide tolerance are scarce. Here, we show that long-living honey bee workers, *Apis mellifera*, are less susceptible to the neonicotinoid thiamethoxam than short-lived workers, and that susceptibility was further reduced when workers were acclimatized under colony compared to laboratory conditions. Following an OECD protocol, freshly-emerged workers were exposed to thiamethoxam in summer and winter and either acclimatized within their colony or in the laboratory. Mortality and sucrose consumption were measured daily and revealed that winter workers were significantly less susceptible than summer workers, despite being exposed to higher thiamethoxam dosages due to increased food consumption. Disparencies in fat body activity, which is key for detoxification, may explain why winter bees were less susceptible. Furthermore, colony acclimatization significantly reduced susceptibility towards thiamethoxam in winter workers likely due to enhanced protein nutrition. Brood absence and colony environment seem to govern workers’ ability to cope with pesticides, which should be considered in risk assessments. Since honey bee colony losses occur mostly over winter, long-term studies assessing the effects of pesticide exposure on winter bees are required to better understand the underlying mechanisms.

## Introduction

Intensified agriculture has caused for the simplification of floral landscapes and loss of wild plants (Gonthier et al. 2014), as well as an increased level of environmental pollution in the form of agricultural and horticultural pesticides (Woodcock et al. 2016). Hardly surprising, invertebrate biodiversity has been severely negatively affected by the direct consequences of intensified agriculture (Dudley and Alexander 2017). Concern arises particularly from the increased reports of declines and losses of wild bee species that provide pollination services, which are indispensable to ecosystem functioning and human food security (Scudder 2017; Sanchez-Bayo and Wyckhuys 2019; Zattara, Aizen 2021). Concerns are further amplified by the annual global high losses of honey bee colonies over the past decades (Neumann and Carreck 2010; Requier et al. 2018; Gray et al. 2020; Seitz et al. 2022). While managed Western honey bees, *Apis mellifera*, are indispensable for modern agricultural practices (Aizen and Harder 2009; Ostermann et al. 2021), they too face the ramifications of reduced floral diversity and especially the increased exposure to a wide array of agrochemicals, which has received much attention.

This particularly holds true for neonicotinoid insecticides which are amongst the most frequently used chemicals globally (Klingelhöfer et al. 2022). Targeted to affect the nervous system of pest insects by acting as an agonist of postsynaptic nicotinic acetylcholine receptors (nAChRs) and thereby causing paralyzes and ideally death of a target organism, this class of insecticides has a considerable toxicity to a wide range of invertebrate species (Bass and Field 2018; Matsuda et al. 2020). Due to the systemic properties of some neonicotinoid chemicals (e.g., thiamethoxam), residues are frequently detected in pollen and nectar of blooming crops and neighboring wild floral resources due to run-off from agricultural fields (Goulson 2013), by which they inadvertently become a potential threat to non-target organisms, such as bees (Fairbrother et al. 2014). Indeed, a myriad of studies have revealed lethal (Blacquiere et al. 2012) and sub-lethal effects on consumption behavior (Creswell et al. 2012; Kessler et al. 2015), altering gut microbiota (Liu et al. 2020), or impairing metabolic pathways and detoxification abilities (Fairbrother et al. 2014). Despite the vast number of studies investigating the effects of neonicotinoids on bees, our knowledge on how factors such as seasonality or nutrition may alter susceptibility within a species remains scarce. This is surprising, considering that seasonality is ubiquitous and a strong source of external variation influencing almost all natural systems (Levins 1968).

Organisms living in environments that experience seasonality can be subjected to fluctuating selection on life history traits that may elicit adaptive responses (Varpe 2017). The Western honey bee, *Apis mellifera*, represents an ideal model organism to investigate how susceptibility towards pesticides may vary across seasons. Long-lasting periods of floral dearth (e.g., winters in temperate regions) result in the absence of brood in honey bee colonies. In order to survive such annual fluctuations in selection pressures, long- and short-living worker bees (i.e., several months vs. a few weeks) have evolved due to developmental plasticity (Riley 1985; Smirle and Winston 1987). Despite being morphologically indistinguishable from one another, such short- and long-lived workers differ substantially in physiological and behavioral traits (e.g., immune response (Steinmann et al. 2015). As such traits are often subject to conflicting selection scenarios (Schluter et al. 1991), enhanced survival is likely due to trade-offs with other beneficial yet costly traits (Sheldon and Verhulst, 1996; Flatt and Heyland, 2011). Indeed, such trade-offs have been reported between immune defense and longevity for bumble bees (Moret and Schmid-Hempel 2000) as well between detoxification and longevity in other insects (Flatt and Heyland 2011). Thus, a focus of pesticide effects on short-lived honey bees may involve the risk of underestimating the potential harm to long-lived bees and ultimately the entire colony (Retschnig et al. 2022). Despite exposure scenarios varying across seasons, where summer workers are likely exposed to higher levels of pesticides whilst foraging, winter workers are also subject to exposure via contaminated food storages (e.g., honey and beebread), albeit at lower concentrations (Chauzat et al. 2006; Colding et al. 2016). Subsequently, this conflicting selection scenario in honey bee workers may be a plausible mechanism for the observed increased winter honey bee colony losses, and thus an improved understanding of the life-history trade-offs in workers and their response to pesticide exposure is urgently required.

Besides seasonal and physiological differences, the quantity and balance of nutrients, as well as secondary metabolites, in an insect’s diet can enhance the ability to respond to xenobiotic pressures and thus have beneficial effects on longevity (Simpson and Raubenheimer 2012). For instance, sensitivity towards a toxin can be modulated by the carbohydrate to protein ratio (Deans et al. 2017) and secondary metabolites have been shown to stimulate the expression of specific detoxification enzymes, increasing a bee’s tolerance (Johnson et al. 2012). Adult honey bee workers typically consume pollen within the first few days of adulthood (Pernal and Currie 2000), whereby the lack of pollen access during these first days may elicit substantial differences in an individual’s ability to respond to xenobiotic exposure. Current regulatory chronic toxicological risk assessment studies omit the chronic feeding of contaminated pollen and nectar when testing effects on honey bees for 10 days (e.g., OECD guidelines 245), yet comparative data could lead to improved risk assessment strategies for any given chemical substance.

Using the OECD 245 guidelines for chronic toxicity (OECD 2017), we here compared the susceptibility of short-lived (i.e., summer) and long-lived (i.e., winter) worker honey bees, *A. mellifera*, to the neonicotinoid thiamethoxam. In addition, to access how pollen availability under natural colony conditions can impact susceptibility, bees were acclimatized either under colony or laboratory conditions for three days prior to being exposed. Considering previous studies (Baines et al. 2017; Barascou et al. 2021), we hypothesized that long-lived (i.e., winter) workers will be significantly more susceptible compared to short-lived (i.e., summer) workers, resulting in an increased mortality. Further, workers acclimatized under colony conditions will have a decreased susceptibility to the neonicotinoid comparted to workers acclimatized in the laboratory.

## Material and methods

### Experimental design

Between May and December 2022, the study was conducted at the Institute of Bee Health, Bern, Switzerland, using three local, non-related queenright *A. mellifera* (L.) colonies managed using Best Management Practices including an oxalic (2.7%) acid treatment in the previous winter (2021) and a formic (85%) acid treatment in August 2022 against *Varroa destructor* mites (Dietemann et al. 2013). To warrant comparability of our data across seasons (i.e., summer and winter), the identical three colonies were used throughout the entire study to source newly emerged workers and so minimalize genetic variation (Sandrock et al. 2014). Mite infestation levels were quantified for each colony (01 May and 01 November 2022) using the sticky bottom board and individual worker brood cell infestations (Dietemann et al. 2013) and revealed no significant difference in infestation. Furthermore, to ensure similar colony strength, Liebefeld assessments were performed before sourcing experimental workers in summer (early June) and winter (early November; Delaplane et al. 2013). Irrespective of the season, colony strength assessments revealed no significant differences amongst the three colonies. Summer bees were obtained in early June when all three colonies had ample brood (i.e., at least four frames of brood). Whereas the winter bees were obtained in early November by collecting the last brood patches found in each colony. After removing these frames of brood in November, colonies were inspected in early December to ensure the colonies were still brood free (which they were) and thus ensuring the obtained workers in November were most certainly long-lived “winter” bees.

### Insecticide exposure and preparation of solutions

Chronic oral toxicity tests were performed following the OECD 245 guidelines (OECD 2017). In brief, to obtain sufficient workers of the same known age cohort, we selected brood frames containing workers that were within 24 h of emergence by uncapping sealed brood cells and insepcting adult coloration (Human et al. 2013). These frames were then removed from the colony and transferred to a laboratory incubator and maintained in complete darkness at 34.5 °C and 60% relative humidity (RH) (Williams et al. 2013). Upon emergence, each experimental worker was visually examined for clinical symptoms of disease, physical abnormalities and/or the presence of the parasitic mite *V. destructor* (Williams et al. 2009; Dietemann et al. 2013). To avoid confounding factors, individuals displaying any abnormality (i.e., deformed wings) or *V. destructor* infection were excluded from the experiment. Newly-emerged workers from all three colonies were randomly allocated to standard hoarding cages (200 [cm^3^]; Straub et al. 2016) consisting of 20 individuals per cage. Cages were maintained in complete darkness at 30 °C and 60% RH, and given 50% [w/v] sucrose solution ad libitum via a 5 mL syringe (Codan Medical AG, Switzerland) to provide a carbohydrate energy source (Williams et al. 2013). To ensure that the workers were healthy and accustomed to their cages and feeding system prior to being exposed to their respective treatment groups, individuals were maintained in their cages for the first 72 h (i.e., acclimatization phase). Mortality was monitored daily during the acclimatization phase and to avoid transmission of potential disease, cages with dead individuals were excluded from the experiment so that at the beginning of the treatment exposure period all cages consisted of 20 workers.

In total, eight thiamethoxam treatments (i.e., feeding solutions) of decreasing concentrations were used, as well as a positive (i.e., dimethoate), and negative control (i.e., only aqueous sucrose solution (50% [w/v]). To produce the thiamethoxam feeding solutions 10.9 mg of thiamethoxam (CAS-153719-23-4, 99% purity, Sigma-Aldrich, UK) was diluted in 10.9 mL of pure acetone to obtain a super stock solution of thiamethoxam at a concentration of 1 mg mL^–1^ in pure acetone. The super stock solution was then used to produce a stock solution containing 1000 ng ml^−1^ of thiamethoxam by adding 1 mL of the super stock solution to 999 mL of aqueous sucrose solution (50% [w/v]). This stock solution (consisting of aqueous sucrose solution (50% [w/v] and 1000 ng thiamethoxam dissolved in 1 mL acetone) was used to obtain our highest thiamethoxam feeding solution (i.e., 1000 ng mL^−1^ treatment group). Then, by using a standard dilution series, the stock solution also acted as the basis for the subsequent thiamethoxam feeding solutions (i.e., 500, 100, 40, 20, 10, 4.5, and 1.5 ng mL^−1^ treatment groups). Subsequently, the 500 ng mL^−1^ feeding solution was made by taking 500 mL of the stock solution and adding it to 500 mL of aqueous sucrose solution (50% [w/v]). The 100 ng mL^−1^ feeding solution was then produced by taking 200 mL of the 500 ng mL^−1^ feeding solution and adding it to 800 mL of aqueous sucrose solution (50% [w/v]), and so forth. The choice of thiamethoxam concentrations aimed to cover both a field-realistic range (i.e., 1.5–10 ng g^−1^ (Decourtye and Devillers 2010; Blacquière et al. 2012)) as well as cover official ecotoxicological guidelines that would ensure the calculation of a lethal daily dosage (LDD_50_) (EFSA 2013; OECD 2017). Following the same method as previously described for the thiamethoxam, the positive control (i.e., dimethoate (CAS-153719-23-4, 99% purity, Sigma-Aldrich)) feeding solution was produced by first preparing a super stock solution at 1 mg mL^–1^ in pure acetone and then adding 1 mL of the super stock dimethoate solution to 999 mL of aqueous sucrose solution (50% [w/v]) to obtain a 1000 ng mL^−1^ dimethoate feeding solution. To account for the acetone used to prepare the pesticide feeding solutions, the control feeding solution was prepared following the same procedure as previously described for thiamethoxam and dimethoate, however without adding a pesticide to the control super stock solution. Irrespective of the treatment group, the acetone concentration in all feeding solutions made up for less than 5% of the volume. To avoid chemical degradation, all vials were kept in complete darkness at 4 °C and wrapped in tin foil. Solutions were freshly prepared upon usage. The stock solutions were newly prepared before the beginning of the summer and winter trials and sucrose solution feeders were replaced every 72 h. To confirm the pesticide preparation was adequately performed, high-performance liquid chromatography (HPLC; Agilent 1290 Infinity II) coupled with mass spectrometry (MS/MS; Aligent 6495C tandem quadrupole) was conducted on the summer and winter feeding solutions of the control and 1.5 ng mL^–1^ thiamethoxam treatment groups following Schaad et al. (2023). The detected residues confirmed our thiamethoxam concentrations for summer and winter thiamethoxam (i.e., 1.48 and 1.51 ng mL^−1^) and control feeding solutions (i.e., below limit of detection (LOD) 0.4 ng mL^−1^) and thus verifying our solution preparation.

### Consumption and survival assessment

After the acclimatization phase, cages (*N* = 45 per season) were randomly assigned to either the thiamethoxam treatment group (i.e., 1,000, 100, 40, 20, 10, 4.5, and 1.5 ng mL^−1^) or to the positive (i.e., dimethoate) or negative (i.e., control) treatment group. Each treatment consisted of five replicates (i.e., *N* = 5 per treatment and season; each cage with 20 workers; *N* = 100 workers per treatment) and workers were fed ad libitum feeding solution according to the treatment group. To assess the daily consumption of an individual worker, feeding solution volumes of each cage were assessed at the beginning and after three days, or whenever an individual worker died within the three days, as recommended in the OECD 245 guideline (OECD, 2017). The difference between end and start volume was then divided by the total number of workers present in a cage, to determine daily consumption [mL bee^−1^ day^−1^]. This enabled the calculation of individual daily thiamethoxam- or dimethoate-exposure [ng bee^−1^ day ^−1^]. In addition, to enable precise measurements, evaporation was accounted for by using five empty cages containing a syringe with control feeding solution. The daily average evaporation rate was then subtracted from each daily individual consumption value. Mortality was monitored daily. Dead bees were counted and removed from their respective cages. Individuals that survived the 10-day exposure period were removed from their cages. The identical protocol as described above was applied for both June (i.e., summer) and November (i.e., winter), respectively. However, in contrast to the summer trial, the winter trial had an increased sample size (i.e., *N*_winter_ = 1140; *N*_summer_ = 1000 workers in total) due to an additional thiamethoxam treatment group (i.e., 500 ng mL^−1^) and all winter treatment groups, with the exception of the 500 and 1000 ng mL^−1^ thiamethoxam and dimethoate groups, consisted of six cages (i.e., *N* = 120 workers) rather than five cages (i.e., *N* = 100 workers).

### Comparing laboratory vs. colony acclimatization phase

To test whether the environment in which a bee stayed during the first days (i.e., 72 h) of its life could affect consumption behavior and susceptibility, we additionally marked 350 newly emerged winter bees that were not used for the chronic toxicity test with non-toxic acrylic paint following Straub et al. (2021). These individuals were then placed back in one of the three maternal hives which was completely free of brood (i.e., no eggs, larvae, or capped brood) in which they stay for 3 days (i.e., colony acclimatization phase). It was essential that the colony was brood-free as we assumed this would trigger a physiological response making the bees long-lived ‘winter’ bees (Knoll et al. 2020). After 72 h, marked workers were counted to determine mortality rate and 120 individuals were removed from the colony, brought to the laboratory, and randomly assigned to either the 0 (*N* = 60; i.e., control), 4.5 (*N* = 60) or 40 ng mL^−1^ (*N* = 60) thiamethoxam treatment group. These concentrations were chosen to challenge the detoxification abilities of individual bees at both a field-realistic (i.e., 4.5 ng mL^−1^) as well as an extreme (i.e., 40 ng mL ^− 1^) scenario (Zioga et al. 2020; Zioga et al. 2023). Following the previously described methods, bees were exposed to their respective treatment groups for 10 days and consumption behavior, exposure as well as daily mortality data were collected.

### Data curation and analyses

All statistical tests were performed using STATA16 (StataCorp (2017)), while statistical figures were created using NCSS v.12 (NCSS (2020)). All outcome variables (i.e., daily consumption [mL], daily exposure [ng bee^−1^ d^−1^], and mortality [%]) were visually inspected using quantile-quantile plots as well as being tested for normality using the Shapiro–Wilk’s test and the Levene’s test for homogeneity of variances. The subsequent statistical methods were chosen accordingly (see supplementary information (SI) Table 1). To evaluate a potential connection between explanatory variables and dependent variables, linear regression mixed-effects models (LMMs) were applied. Multilevel generalized linear (regression) models (GLMMs) with random intercept were fitted using the STATA function *meglm*, wherein bees were considered independent units, “season” (i.e., summer or winter), “acclimatization” (i.e., laboratory or colony), “exposure” (i.e., pesticide exposure or non-exposed), and ‘concentration’ (i.e., 0–1000 ng mL^−1^) were included as the explanatory (fixed) terms and whenever applicable the fix covariate ‘age’ as well as the random effect ‘cage’ was incorporated. For each multiple regression model, a stepwise backward elimination approach was applied to determine the mode of best fit (Wiegand, 2010). Best fit models were chosen by comparing every multilevel model with its single-level model counterpart using both a likelihood ration (LR) test as well as the Akaike information criterion (AIC) using the function *lrtest* and *estat ic*, respectively. Whenever appropriate, the means ± the standard error (SE) (adjusted for distribution) are given in the text and are further provided in SI Table 1 along with the results from the statistical tests. In addition, summary statistics (i.e., sample size, mean, standard error, as well as the upper and lower 95% confidence interval) for all measured variables are provided in SI Table 2.

Daily consumption [mL] and daily exposure [ng mL^−1^] were non-normally distributed (Shapiro-Wilk’s test, *p* < 0.05) and were therefore modeled with a GLMM following either error Gamma or Poisson distribution. Following Ritz et al. (2015) we used a fitted dose-response model based on a log-logistic regression analysis to determine the 10-day oral Lethal Daily Dosage (LDD)_50_ (expressed in ng bee^−1^ day^−1^) values with 95% confidence limits. The survival analyses for the acclimatization phase data were performed using the *mestreg* function for multilevel survival models (Cleves, 2002). Survival was calculated by using cumulative survival rates [%] after 4 days for each treatment. Survival curves (Kaplan Meier plots) and smooth estimated hazard rate plots with 95% confidence intervals (CI) were used to visually display the survival data. Post-hoc comparisons amongst treatment groups for all variables were conducted using pairwise Bonferroni multiple comparisons test (*bmct*), using the function *mcompare()* and option *bonferroni*, whenever necessary (Mitchell 2012).

## Results

Across both seasons, dimethoate exposure led to a significantly increased mortality rate when compared to the controls (*z* = −48.49, *p* < 0.001). While control mortality over the 10 days was below 5%, dimethoate lead to a 100% mortality after nine days whereby 50% mortality was reached after 4 days (SI Fig. 1).

### Consumption and exposure

**Fig. 1:**
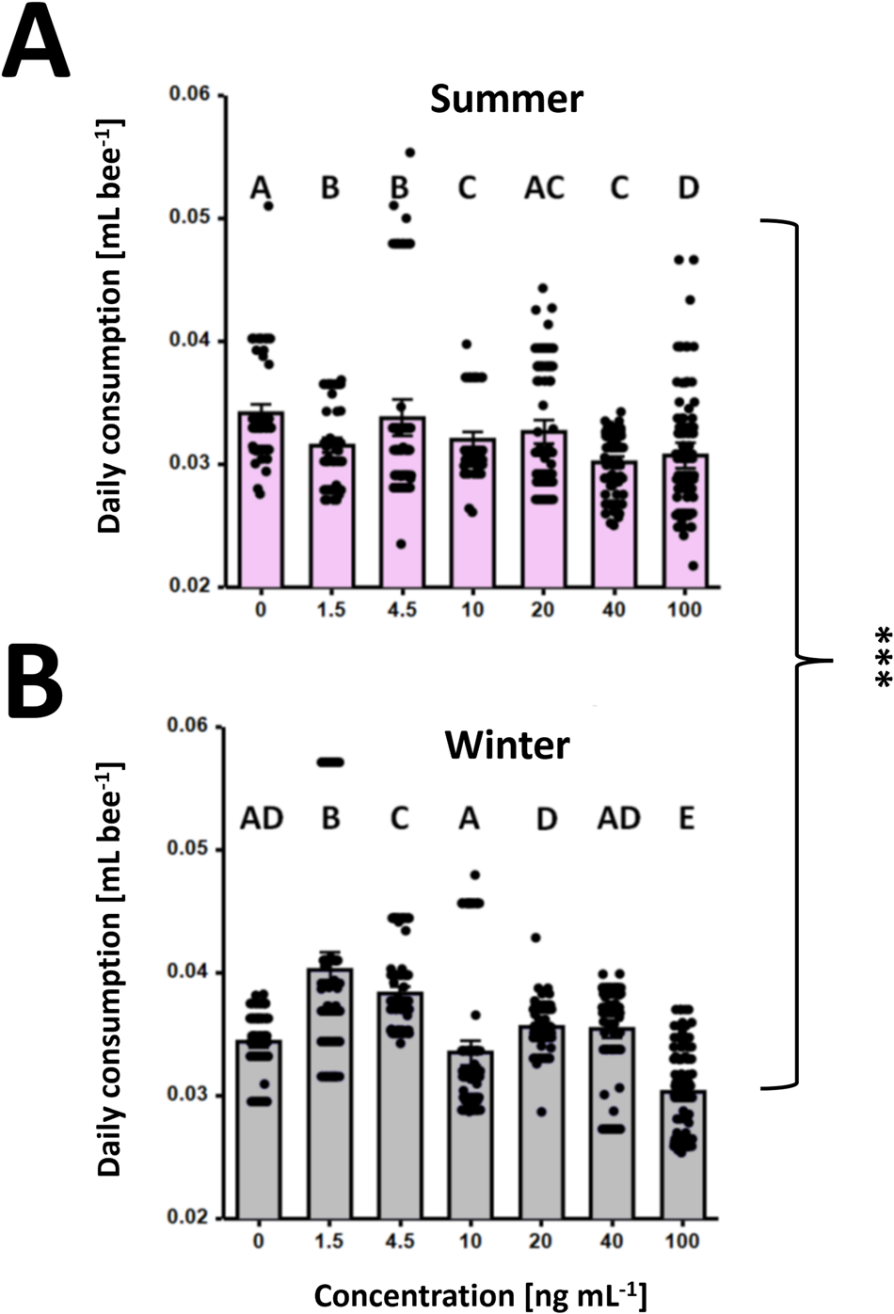
Daily sucrose solution consumption rates for (**A**) summer and (**B**) winter honey bee female (worker) bees, *Apis mellifera*, exposed to increasing thiamethoxam concentrations. Significant differences in consumption were observed amongst the different tested concentrations for both seasons (*p* < 0.05), as well as an overall increase in consumption during winter compared to summer (*p* < 0.001). The bar charts show both means (boxes) and standard errors (horizontal black bars and points) with the violet and gray bars representing summer and winter bees, respectively. Significant differences amongst concentrations (i.e., *p* values < 0.05) are indicated by letters (e.g., A, B, C), whereas a significant difference among the daily consumption of summer and winter bees (*p* values < 0.001) is indicated by asterisks (***)

**Fig. 2:**
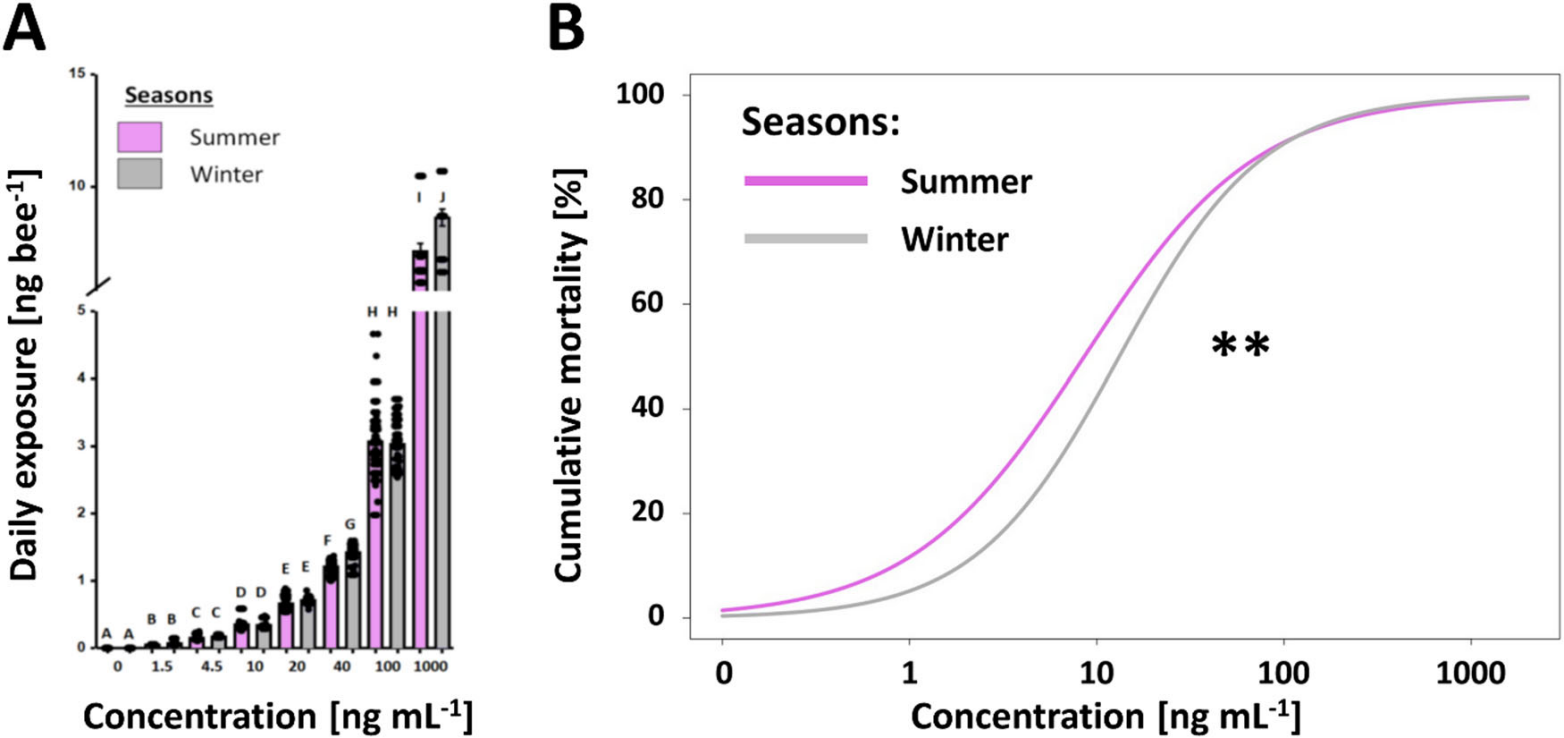
Daily thiamethoxam exposure rates and the acute oral *Lethal Daily Dosage*_50_ (*LDD*_50_) curves for summer and winter female (worker) honey bees, *Apis mellifera*. **A** Increasing concentrations of thiamethoxam resulted in a significant increase in daily exposure [ng bee^−1^] (*p* < 0.001), wherein difference between seasons of bees from the same treatment group (i.e., concentration) were observed (*p* < 0.05). The bar charts show both means (boxes) and standard errors (horizontal black bars and points) with the violet and gray bars representing summer and winter bees, respectively. Significant differences amongst concentrations (i.e., *p* values < 0.05) are indicated by letters (e.g., A, B, C). **B** Fitted dose-response curves based on a log-logistic regression analysis were used to determine the acute oral mortality rate for both summer (solid violet line) and winter workers (solid grey line) for increasing thiamethoxam concentrations. A significant difference in the dose-response curves was revealed (*p* < 0.01) as depicted by the asterisk (**)

Irrespective of season and exposure, increasing age significantly reduced sucrose consumption resulting in a negative correlation between daily consumption and age (*z* = −3.87, *p* < 0.001). Further, a seasonal effect was found, as daily consumption in winter bees was significantly higher compared to summer bees (*z* = 14.75, *p* < 0.001; Fig. 1). In summer, a significant dose-dependent effect was observed (*z* = −14.7, *p* < 0.001), resulting in a negative correlation between increasing thiamethoxam concentration and daily consumption (Fig. 1A). With the exception of the 20 ng mL^−1^ (*p* = 1.0; 32.65 ± 0.48 μL ± SE), summer control daily consumption differed from the remaining thiamethoxam treatments (*bmct*; all *p*’s < 0.039; 31.65 ± 0.62 μL± SE), where controls’ consumed ~7.5% more (34.15 ± 0.37 μL ± SE). Conversely, a non-linear effect was observed among the winter bees when comparing the daily consumption across treatment groups. Interestingly, individuals of the 1.5 ng mL^−1^ (40.21 ± 0.75 μL ± SE) and 4.5 ng mL^−1^ (38.28 ± 0.29 μL± SE) treatment groups (both *p*’s < 0.001) consumed significantly more than the controls (34.41 ± 0.23 μL ± SE), resulting in a 17% and 11% increase, respectively (Fig. 1B). In contrast, the daily consumption of the 10 ng mL^−1^ (33.50 ± 0.51 μL± SE), 20 ng mL^−1^ (35.56 ± 0.16 μL ± SE), and 40 ng mL^−1^ (35.45 ± 0.35 μL± SE) treatments did not significantly differ from the controls (all *p*’s > 0.77; Fig. 1B). However, the 100 ng mL^−1^ (30.29 ± 0.30 μL ± SE) treatment group revealed a significant negative effect on daily consumption (*p* < 0.001), revealing a 12% decrease in consumption. Subsequently, winter bees were on average exposed to 14% more thiamethoxam (2.05 ± 1.16 ng ± SE) compared to summer bees (1.80 ± 0.97 ng ± SE; *z* = 4.81, *p* < 0.001; Fig. 2A). This difference was most apparent in the 40 ng mL^−1^ (winter = 1.42 ± 0.01 ng bee^−1^ day^−1^ ± SE; summer = 1.21 ± 0.01 ng bee^−1^ day^−1^ ± SE) and 1000 ng mL^−1^ (winter = 8.62 ± 0.19 ng bee^−1^ day^−1^ ± SE; summer = 7.12 ± 0.17 ng bee^−1^ day^−1^ ± SE) treatment groups, where winter bees were exposed to 17.41% and 21% more thiamethoxam than summer bees, respectively (Fig. 2A).

### Survival and 10-day LDD_50_

Thiamethoxam exposure revealed a negative dose-dependent effect on both winter and summer worker survival (*z* = 10.77, *p* < 0.001), whereby increasing thiamethoxam exposure negatively correlated with survival. In addition, a seasonal effect on survival was observed, where overall survival rates were significantly higher for winter workers compared to summer workers (*z* = 3.32, *p* = 0.001). The seasonal effect became most apparent when comparing individual treatments by season (e.g., control summer vs control winter; SI Fig. 2). The winter control, 1.5, 40, and 100 ng mL^−1^ thiamethoxam treatment groups significantly differed from their respective summer treatments (all *z*’s < −2.01, all *p*’s < 0.44; SI Fig. 2A–D), resulting in a 7.6%, 11%, 32% and 44% increased survival of the winter compared to summer workers, respectively. Subsequently, the *LDD*_50_ for summer workers (8.56 ± 6.9–10.2 ng bee^−1^ day^−1^ ± 95% CI) was significantly lower compared to the winter workers (13.18 ± 11.2–15.2 ng bee^−1^ day ^−1^ ± 95% CI; *p* = 0.005), reflecting 35% increased susceptibility towards thiamethoxam (Fig. 2B).

### Acclimatization effect

**Fig. 3:**
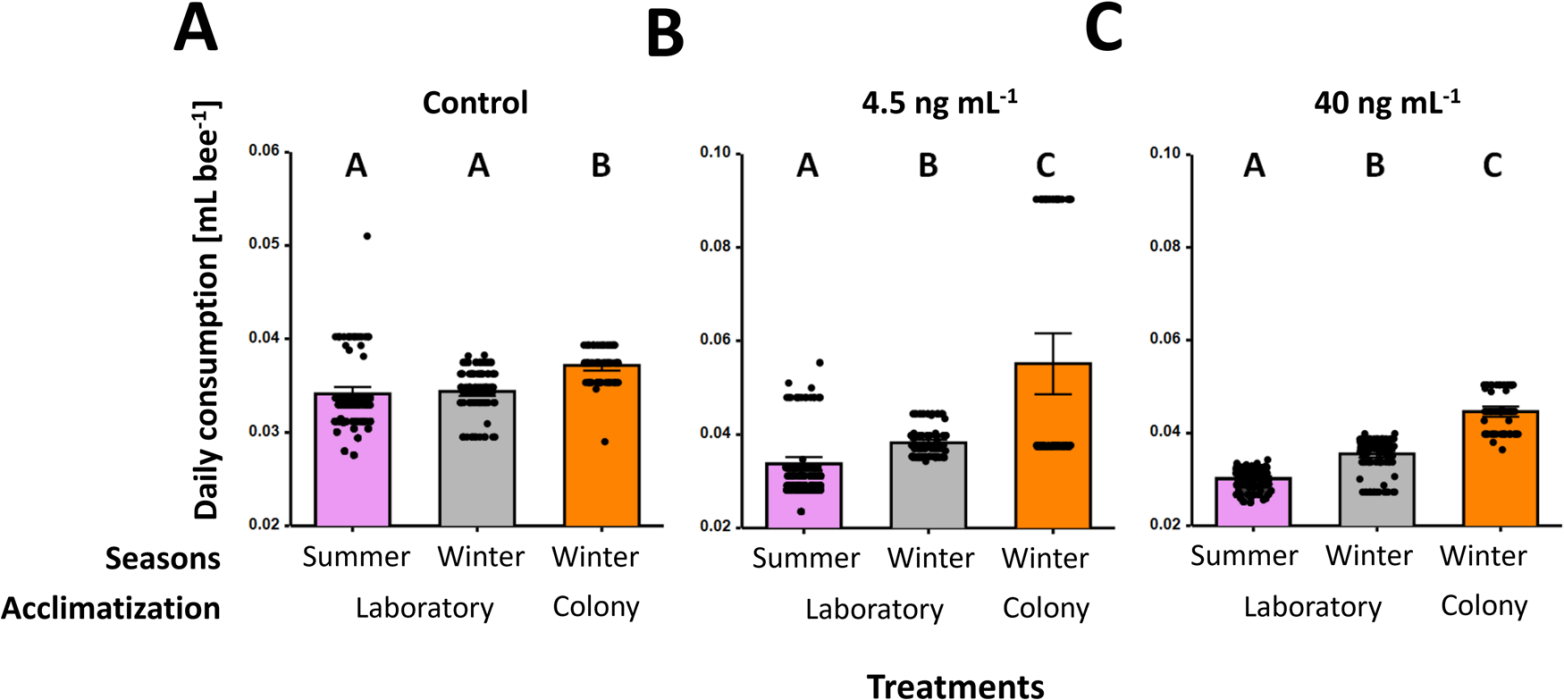
Seasonal and acclimatization effects on consumption behavior of female honey bee (workers) *Apis mellifera*, exposed to vary concentrations of thiamethoxam. Daily consumption of summer and winter bees as well as colony acclimatization workers for (**A**) control (**B**) 4.5 ng mL^−1^ and (**C**) 40 ng mL^−1^ thiamethoxam treatment groups. The bar charts show both means (boxes) and standard errors (horizontal black bars and points) with the violet, gray and orange bars representing summer laboratory acclimatized, winter laboratory acclimatized, and winter colony acclimatized bees, respectively. Significant differences amongst concentrations (i.e., *p* values < 0.05; Bonferroni corrected) are indicated by letters (e.g., A–C)

The colony acclimatization phase revealed a significant positive effect on daily consumption (*z* = 10.86, *p* < 0.001). Irrespective of the treatment, winter colony acclimatized workers (45.65 ± 5.21 μL ± SE) consumed significantly more than the laboratory acclimatized summer (32.71 ± 1.26 μL ± SE) and winter (36.05 ± 1.16 μL ± SE) workers, resμLting in a 28% and 21% increase, respectively (all *z*’s > 2.93, *p*’s < 0.003). With respect to the treatment groups (i.e., control, 4.5 and 40 ng mL^−1^), the control colony acclimatized workers (37.19 ± 0.26 μL ± SE) consumed significantly more than the laboratory acclimatized summer (34.15 ± 0.37 μL ± SE) and winter (34.41 ± 0.23 μL ± SE) workers (both *p*’s < 0.001), reflecting in a 7.8% increased consumption of the colony acclimatized workers (Fig. 3A). However, no significant difference was observed between the summer and winter laboratory workers (*p* = 0.41). Likewise, across the 4.5 ng mL^−1^ exposed workers, the colony acclimatized workers (55.15 ± 3.24 μL ± SE) consumed significantly more than the laboratory acclimatized workers (both *p*’s < 0.001; Fig. 3B). Furthermore, the summer laboratory acclimatized workers (33.80 ± 0.75 μL ± SE) consumed significantly less than the laboratory acclimatized winter workers (38.28 ± 0.29 μL ± SE) (*p* = 0.01; Fig. 3B). The identical pattern was observed in the 40 ng mL^−1^ treatment group, where colony acclimatized winter workers (44.60 ± 0.58 μL ± SE) consumed significantly more than the laboratory acclimatized summer (30.20 ± 0.24 μL ± SE) and winter (35.45 ± 0.35 μL ± SE) workers (both *p*’s < 0.001 Fig. 3C), and the lowest consumption was observed in the summer (30.20 ± 0.24 μL ± SE) laboratory acclimatized workers (*p* < 0.001; Fig. 3C). Consequently, due to the higher daily consumption, the colony acclimatized insecticide treatment workers were exposed to significantly more thiamethoxam compared to their respective laboratory acclimatized treatments (*z* = 2.18; *p* < 0.03). Therefore, in the 4.5 ng mL^–1^ treatment groups, colony acclimatized winter workers (0.25 ± 0.01 ng bee^−1^ day^−1^ ± SE) were on average exposed to 40% and 32% more thiamethoxam than the laboratory acclimatized summer (0.15 ± 0.003 ng bee^−1^ day^−1^ ± SE) and winter (0.17 ± 0.001 ng bee^−1^ day^−1^ ± SE) workers, respectively. Likewise, in the 40 ng mL^–1^ treatment groups the colony acclimatized winter workers (1.78 ± 0.02 ng bee^−1^ day^−1^ ± SE) were exposed to 32% and 20% more thiamethoxam than laboratory acclimatized summer (1.21 ± 0.009 ng bee^−1^ day^−1^ ± SE) and winter (1.42 ± 0.014 ng bee^−1^ day^−1^ ± SE) workers, respectively.

**Fig. 4:**
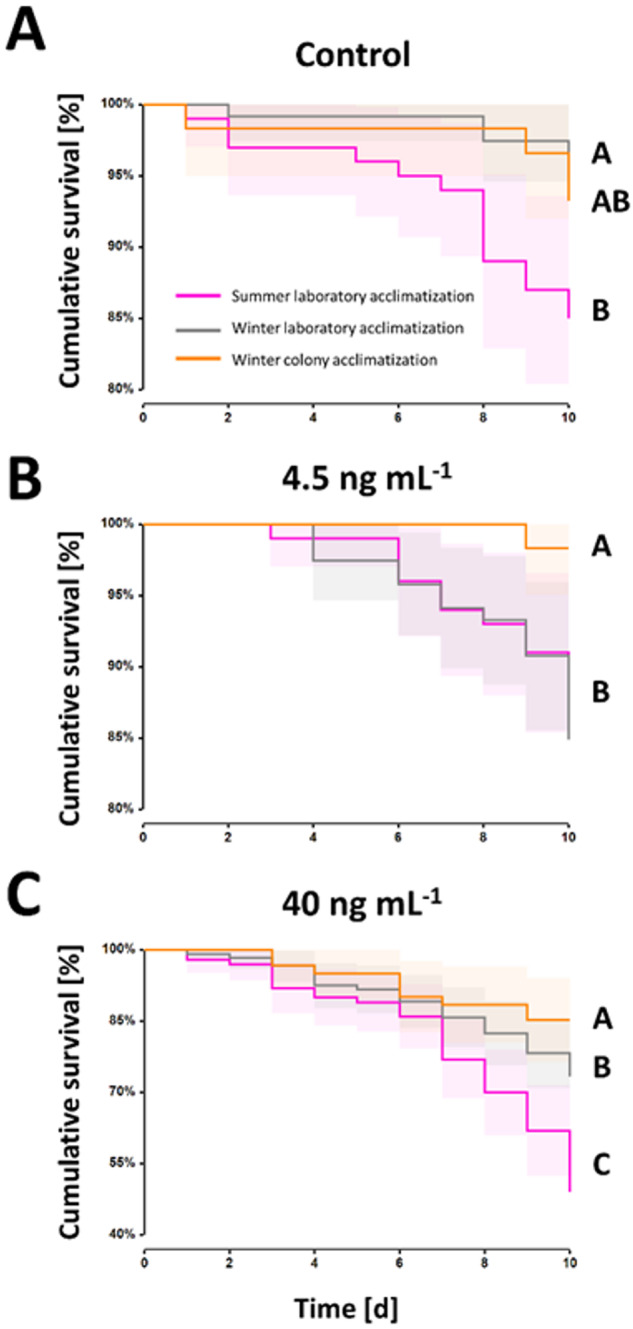
Seasonal and acclimatization effects on survival in female (worker) honey bees, *Apis mellifera*, exposed to varying concentrations of thiamethoxam. CumuLative survival rates were visualized using Kaplan-Meier survival curves. Both seasonal and acclimatization had a significant effect on the survival rates of the bees (*p* < 0.05) for (**A**) 0 (i.e., controls), (**B**) 4.5 ng mL^−1^ and (**C**) 40 ng mL^−1^ treatment groups. The violet line represents the cumuLative survival of the laboratory-acclimatized summer workers; the gray line represents the cumulative survival of the laboratory acclimatized winter workers, whereas the orange line depicts the cumulative survival of the colony-acclimatized winter workers. The shaded areas around the lines represent the 95% confidence intervals. All significant differences (i.e., *p* < 0.05) are indicated by capitalized letters (i.e., A–C)

Colony acclimatization had a significant positive effect on survival (*z* = 2.41, *p* = 0.02). Furthermore, a significant seasonal effect was observed (*z* = 2.09; *p* = 0.04), where summer workers revealed a lower survival rate when compared to winter workers. Across the control treatments, no significant difference was observed between the colony and laboratory acclimatized winter workers (94 ± 92–96 survival [%]; *p* = 1.0; Fig. 4A), however, a significantly different lower survival was revealed for the laboratory acclimatized summer workers (85 ± 78–82 survival [%]) when compared to their winter counterparts (*p* = 0.04, Fig. 4A). Therefore, winter workers on average had a 9.6% increased survival rate. The same pattern was observed in the 4.5 ng mL^–1^ treatment group (Fig. 4B), where winter worker survival (84 ± 78–91 survival [%]) was significantly increased compared to the summer worker survival (89 ± 82–95 survival [%]; *p* = 0.003), however no significant difference was found when comparing to the colony acclimatized workers (98 ± 95–100 survival [%]; *p* = 1.0; Fig. 4B). Further, survival was significantly increased in the colony acclimatized workers (98 ± 95–100 survival [%] ± 95% CI) when compared to the laboratory acclimatized summer workers (89 ± 82–95 survival [%]; *p* = 0.02), resμLting in a 9.2% increase (Fig. 4C). Across the 40 ng mL^–1^ treatment groups, the colony acclimatized workers (85 ± 76–94 survival [%]) had the highest survival rate which significantly differed from the laboratory acclimatized winter (73 ± 65–81 survival [%]) and summer (49 ± 39–59 survival [%]) worker survival, reflecting an increase of 14.1% and 42.4%, respectively (Fig. 4C). The laboratory acclimatized summer workers revealed the lowest survival rate across the treatment groups (*p* = 0.003, Fig. 4C).

## Discussion

The data show that seasonality and bee acclimatization can govern the susceptibility of honey bee workers to a frequently applied agrochemical. Although being exposed to higher dosages of the neonicotinoid thiamethoxam — due to increased consumption — the LDD_50_ of winter workers was 35% higher compared to summer workers, suggesting an enhanced tolerance. Further, colony acclimatization reduced susceptibility towards thiamethoxam in winter workers compared to laboratory acclimatized bees — likely due to enhanced protein nutrition. These novel findings may be attributed to seasonal variation in detoxification abilities as well as an adequate diet which may positively affect the abilities of workers to metabolize and cope with xenobiotic exposure. By increasing the probability of individual survival, enhanced detoxification abilities in long-lived honey bee workers may reflect an adaptive trait necessary to ensure that sufficient worker populations are maintained during a highly vulnerable phase of the honey bees colony life-cycle (i.e., long brood-free periods). Additional long-term studies, ideally monitoring survival over several months, are nevertheless required to improve our understanding of how pesticide exposure may affect individual workers as well as their ability to perform essential behaviors (e.g., thermoregulation) during brood-free periods.

Our data confirm previous studies showing honey bee consumption behavior can vary across seasons (Alburaki et al. 2022). While it remains speculative as to why, the observed effect may be attributed to seasonal phenotypic traits including varying hormone titerss, altered metabolic rates, differing immune capacities, and/or differences in fat body content (Smirle and Winston 1987; Haszonits and Crailsheim 1990; Harris and Woodring 1992; Huang and Robinson 1995; Steinmann et al. 2015; Lee et al. 2022). Such differences may concur with varying energetic demands causing for the observed difference in daily sucrose consumption in summer and winter workers. An additional plausible explanation for the apparent varying nutritional needs may be associated with the microbiota which plays a key function in digesting of nutrition such as carbohydrates (Engel et al. 2012). Gut microbiota of an adult honey bee are acquired and developed via contact with hive products, consumption of beebread and honey as well as through trophallactic interactions with older bees (Powell et al. 2014). As known for a wide range of taxa, including humans (Smits et al. 2017), recent studies in honey bees have shown that the gut microbiota can also vary across seasons (Ludvigsen et al. 2015; Kešnerová et al. 2020). Such seasonal gut microbiota changes may have affected the digestive and metabolic abilities of worker bees, thus altered nutritional demands, and subsequently triggered different consumption behavior. Besides being key for digestion, a sound gut microbiota can elevate sucrose sensitivity as well as enable a more efficient metabolism within the intestine (Flint et al. 2012; Zheng et al. 2017). The colony acclimatized bees most certainly had a distinct microbiota compared to their laboratory counterparts that may have resulted in an enhanced responsiveness and metabolism of sucrose solution, thereby explaining the observed increased daily consumption.

A further seasonal effect was observed when comparing the effects of thiamethoxam on consumption behavior. In line with previous laboratory data (Overmyer et al. 2018), increasing exposure in summer bees revealed a reduced daily intake of sucrose when compared to controls. This is likely explained by either a learned avoidance behavior or attributed to the neurotoxic properties of the tested substance reducing the ability or willingness of the bees to feed (Williamson et al. 2014; Tosi et al. 2016). In contrast, this effect was only observed in the 100 ng mL^–1^ exposed winter workers, whereas the remaining winter bees revealed either no effect or an increase in consumption when compared to their respective controls. Due to the mode of action, neonicotinoids may cause an overexcitation of the nervous system which in return may lead to hyperactivity and subsequently explain the increased consumption (Baines et al. 2017; Tosi and Nieh 2017).

Colony acclimatized intoxicated workers revealed a significantly increased sucrose consumption compared to their respective laboratory-acclimatized counterparts. Again, reasons for this disparity remain elusive but are possibly coupled to the optimal nutritional conditions within the hive, as well as care by nestmates and/or similar factors as previously discussed (i.e., seasonal phenotypes and gut microbiota). Whether the observed effects on consumption behavior will convey to the colony level remains to be tested. Recent studies suggest that chronic colony level exposure via sucrose solution at concentrations as high as 100 ng L^−1^ had no significant negative effect on work consumption (Overmyer et al. 2018; Thompson et al. 2019). The observed laboratory effect may be suppressed under field colony conditions. This may be due to the increased energy demands of workers that are consistently performing in-hive duties (e.g., brood care, cleaning duties, ventilating the hive) as well as the possibility that the provided sucrose solution was simply stored as honey. Irrespective of the underlying mechanism responsible for varying consumption behavior across seasons and acclimatization environments, winter bees and in particular the colony acclimatized workers, where exposed to significantly higher dosages of thiamethoxam. This finding is key for understanding the mortality data as it highlights that seasonal-specific consumption behaviors inevitably result in differing exposure scenarios. Such differences may be relevant when considering lethal concentration values (i.e., LC_50_) as winter bees would inevitable be exposed to higher dosages compared to summer bees. However, given neonicotinoid residues in honey bee hive products are often detected at lower concentrations when compared to nectar and pollen upon which summer bees forage (Thompson et al. 2019; Zioga et al. 2020), the increased consumption may not inflict additional harm. Nevertheless, additional comparative LC_50_ data for honey bees across seasons would be interesting.

To our knowledge, this is the first report comparing chronic LDD_50_ value of summer and winter exposed honey bees to thiamethoxam. The determined LDD_50_ for summer (8.56 ng bee^−1^ day^−1^) and winter (13.18 ng bee^−1^) workers lies clearly above the only previously determined value (i.e., 2.45 ng bee^−1^ day^−1^ (Overmyer et al. 2018). Both genetics and increased temperatures have been shown to affect the susceptibility of bees towards pesticide exposure (Sandrock et al. 2014; Kenna et al. 2023. Therefore, reasons for this disparity may be varying genetics as well as the on average warmer incubator conditions (i.e., 30 °C in this study; ~32.5 °C in Overmyer et al. 2018) under which the bees were kept. Interestingly, our data revealed that the LDD_50_ for winter workers was 35% higher, suggesting a clearly reduced susceptibility in winter compared to summer bees. The evident improved ability to cope with chronic thiamethoxam exposure is further undermined by the fact that winter bees on average were exposed to 14% more thiamethoxam due to the increased daily consumption. The novel data are in line with a previous study by Meled et al. (1998), which showed that winter bees were eightfold less susceptible to an acute combined pesticide exposure scenario compared to summer bees. Reasons for the observed difference in thiamethoxam sensitivity observed in our study may be multi-fold. For instance, winter honey bees are known to have higher fat body content compared to summer bees (Smirle and Winston 1987; Lee et al. 2022). In bees and insects in general, the fat body is key for intermediary metabolism, which consists of two main types of cells: trophocytes and oenocytes (Abdalla et al. 2015; Brejcha et al. 2023). The oenocytes can act in detoxification processes by expressing cytochrome P450 enzymes and NADPH reductase, which are inherent to biotransformation of xenobiotics (Oliveira and Cruz-Landim, 2006; Conceição de Assis et al. 2022). Furthermore, fat body cells are responsible for the expression of Vitellogenin (Vg). Due to Vg’s zinc-carrier properties, it can act in intoxicated bees as an anti-oxidative thereby countering xenobiotic induced oxidative stress (Amdam et al. 2012; Knoll et al. 2020). As the expression of Vg is far higher in winter bees compared to summer bees (Steinmann et al. 2015), and is closely associated with extending bee longevity (Seehuus et al. 2006; Corona et al. 2007), the higher Vg levels may have contributed to the enhanced ability of winter bees to detoxify and tolerate thiamethoxam. Indeed, honey bee queens displayed a higher tolerance to acaricides compared to workers (Dahlgren et al. 2012), likely due to their elevated Vg expression and subsequently enhanced detoxification capacities (Seehuus et al. 2006).

Colony acclimatization further reduced the susceptibility of workers to thiamethoxam exposure, which resulted in a remarkable 42% decreased susceptibility when exposed to a high dosage of the insecticide (i.e., 40 ng mL^−1^). Differences in gut microbiota and varying diets prior to exposure to thiamethoxam likely explain this astonishing effect. A conventional microbiota can enhance the ability to express genes in the intestine that are associated with xenobiotic detoxification, including cytochrome P450s (Wu et al. 2020). Furthermore, access to honey and in particular pollen diversifies the gut microbiota which in return additionally fortifies the detoxification abilities (Huang et al. 2013; Bleau et al. 2020). Moreover, pollen consumption provides beneficial phytochemicals that can trigger diverse metabolic pathways (Magesh et al. 2017; Danihlík et al. 2018; Ardalani et al. 2021), capable of improving the ability of workers to tolerate xenobiotic exposure as shown for the neonicotinoid imidacloprid (Wong et al. 2018; Ardalani et al. 2021). Likewise, honey-specific compounds (e.g., p-coumaric acid or pinocembrin), can upregulate the expression of detoxification genes, including twelve enzymes relevant in metabolizing xenobiotics (Mao et al. 2013). Taken together, an increased fat body content, a mature core of intestinal microbiota, as well as an adequate diet upon emergence appear to be plausible mechanistic explanations for reducing thiamethoxam susceptibility in winter bee, and in particular colony acclimatized bees.

The revealed findings partially contradict our initial hypothesis that long-lived winter honey bees would be more susceptible to pesticide exposure as conflicting selection scenarios would manifest reduced detoxification abilities as a trade-off for increased longevity (Retschnig et al. 2022). It remains to be tested how costly factors governing detoxification actually are and whether having improved detoxification abilities during the winter may be adaptive. For instance, overwintering colonies are more prone to excessive humidity due to less air circulation (Fries 1982), which can favor the colonization of fungi (i.e., mold) on combs as well as stored hive products (e.g., pollen) (Niu et al. 2011). Such mold can present a challenge to bees as it can produce mycotoxins that are known to be toxic and may lead to premature death (Hilldrup and Llewellyn, 1979). Therefore, it appears adaptive for winter bees to have enhanced detoxification abilities compared to summer bees. Furthermore, the routine application of acaricides (e.g., formic acid and oxalic acids) against *V. destructor* (Dietemann et al. 2013) can lead to high residue concentrations which peak in winter (Kast et al. 2021). This may have unintentionally selected for chemically more resistant winter bees (see Tihelka 2018). Nevertheless, pesticide exposure may reflect an unnecessary heavy burden for overwintering bees as the activation of detoxification pathways will undoubtedly lead to elevated metabolic costs concatenating in a diminishment in lipid reserves (i.e., fat body). As vitellogenin, which is essential for extending the lifespan of bees during winter (Amdam et al. 2003), is produced and stored in the fat body, a reduction in fat body content will most likely result in impaired long-term survival. Therefore, survival studies monitoring individuals for more than 10 days are crucial to shed light on how effective the improved detoxification abilities may be to survive an entire winter. Indeed, signs of colony losses are usually not reported early in the winter but rather towards the end of the winter after several weeks or months (Gray et al. 2020; Bruckner et al. 2023a).

Further, bees are seldom exposed to only one environmental stressor and winter bees investing resources to detoxify may come at the expense of being more vulnerable to other stressors such as viruses and parasites. Recent data indeed revealed that winter bees were more susceptible to combined thiamethoxam and *V. destructor* exposure compared to summer bees (Straub et al. 2016). Lastly, while certainly more intricate, monitoring worker survival at the colony level appears crucial as winter bees will need to perform the strenuous task of thermoregulation which is known to be impaired by neonicotinoid exposure in bees (Tosi et al. 2016; Crall et al. 2018). This in combination with a reduced fat body as well as other biotic and abiotic factors (e.g., *V. destructor* parasitism) may increase the probability of honey bee colony loss. While one study has revealed that six-week chronic exposure at concentrations at ≤50 ng mL^−1^ did not significantly affect colony overwintering success (i.e., survival) (Thompson et al. 2019), additional long-term data are nevertheless required - ideally testing exposure at varying timepoints (i.e., spring, summer, and autumn) and over multiple years (Schläppi et al. 2020). Additional data on the possible effects of neonicotinoid exposure on winter bee hypopharyngeal gland (HPG) size development when workers begin rearing new offspring would be interesting. These glands are key for feeding larvae (Hrassnigg and Crailsheim, 1998) and thiamethoxam exposure has revealed to impair gland size in summer workers (Renzi et al. 2016; Karedla et al. 2022; Bruckner et al. 2023b). Reduced HPG size is likely leading to inadequate brood feeding (Hrassnigg and Crailsheim 1998) as well as precocious foraging behavior (Jaycox et al. 1974), which may limit the number of nurses in a colony and thus lead to impaired brood rearing and social behavior (Crall et al. 2018). Such inadvertent effects at an early stage of colony development (e.g., post-winter diapause) could reflect an unsustainable burden for colonies and increase the likelihood of colony loss.

## Conclusion

An improved understanding of how stressor susceptibility varies across different life stages (e.g., larvae and adults) and across time (i.e., seasons) will undoubtedly lead to more informed and robust regulatory protection measures. With respect to toxicological risk assessments, our data suggest that using summer bees likely represents a more conservative assessment of the potential risk imposed by any given substance, especially considering pollen access can significantly enhance tolerance to pesticides (Barascou et al. 2021). Albeit long-term studies assessing the effects of chemicals on long-lived honey bees would be recommendable for future risk assessments, as current OECD guidelines are inappropriate to determine potential long-term negative effects on individual and colony survival. We thus welcome that the latest EFSA guidance documents suggesting incorporating data testing pesticide effects on winter honey bees (EFSA et al. 2023).

## Data accessibility and availability

The complete raw data can be found at the Dryad repository. 10.5061/dryad.8931zcrwv.

### Supplementary Information

Supplementary Information
