# Supplementary material for: Colony environment and absence of brood enhance tolerance to a neonicotinoid in winter honey bee workers, Apis mellifera
Source: Ecotoxicology. 2024 May 23;33(6):608–21. doi: 10.1007/s10646-024-02758-8 (PMC11252217; doi:10.1007/s10646-024-02758-8)
Supplement: Supplementary file 1 — Supplementary Information [file 10646_2024_2758_MOESM1_ESM.docx]

**Title:** Colony environment and absence of brood enhance tolerance to a neonicotinoid in winter honey bee workers, *Apis mellifera*

**Authors:** Manon Bovier^1^, Andrew F. Brown^1,2^, Domenic Camenzind^1^, Lukas Jeker^3^, Gina Retschnig^1^, Peter Neumann^1,3^, Lars Straub^1,4,5^

**Affiliations:**^1^ Institute of Bee Health, Vetsuisse Faculty, University of Bern, Bern, Switzerland

^2^ University of Freiburg, Freiburg, Switzerland

^3^ Swiss Bee Research Centre, Agroscope, Bern, Switzerland

^4^ Faculty of Science, Energy and Environment, King Mongkut's University of Technology North Bangkok, Rayong Campus, Rayong, Thailand

^5^ Centre for Ecology, Evolution, and Behaviour, Department of Biological Sciences, Royal Holloway University of London, Egham, United Kingdom

**Corresponding author:** [lars.straub@unibe.ch](mailto:lars.straub@unibe.ch)

**
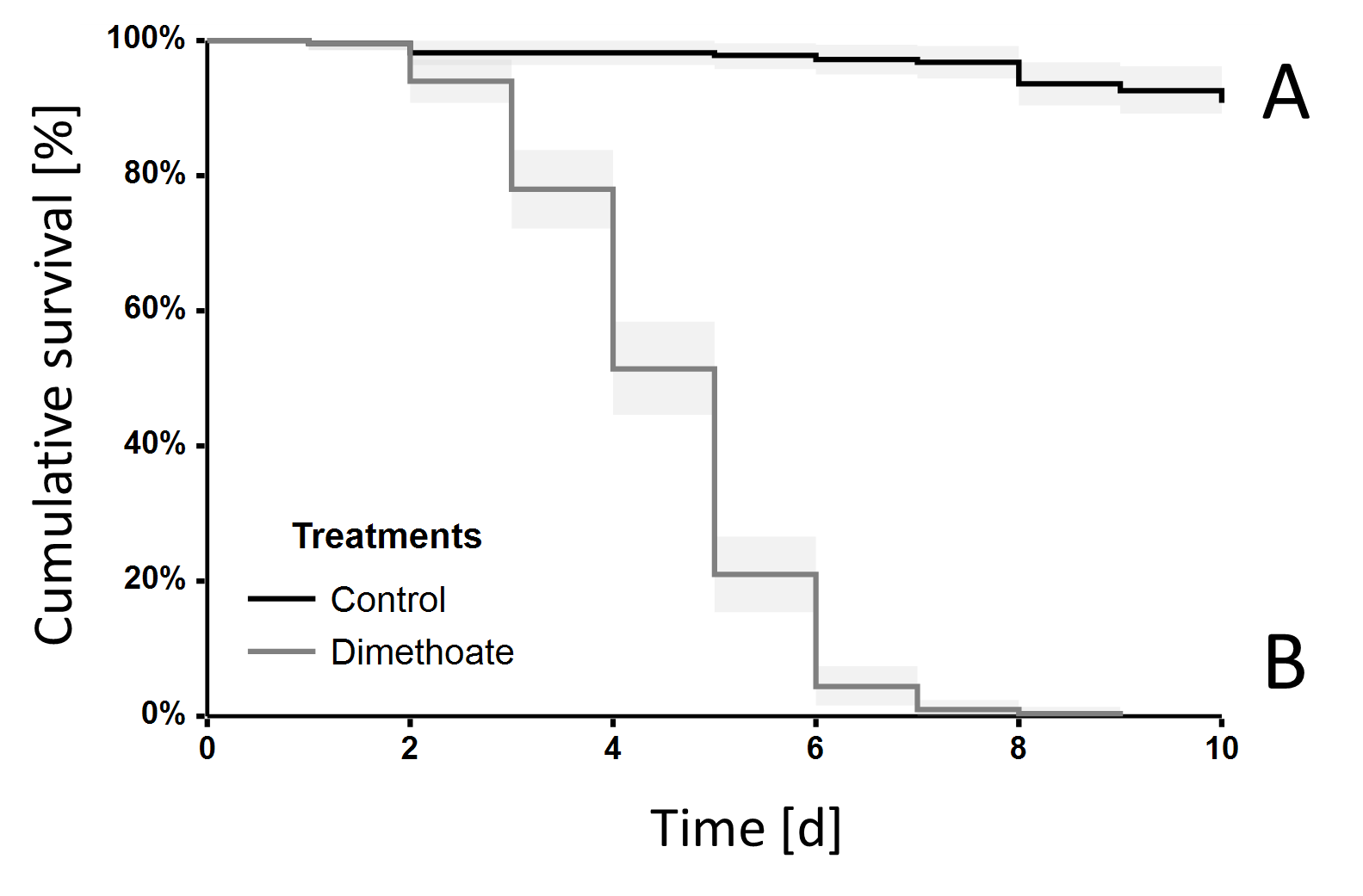
**

**SI Figure 1: Cumulative survival curves for control and dimethoate exposed female (worker) bees, *Apis mellifera***. Kaplan-Meier cumulative survival curves were used to visualize the survival over time. The solid black (i.e., control) and grey (i.e., dimethoate) lines represent the survival rates of the workers exposed to either treatment. Workers from both winter and summer were compiled together for the analysis. Significant differences (i.e., *p* < 0.05) are indicated by the capitalized letters A and B.

**
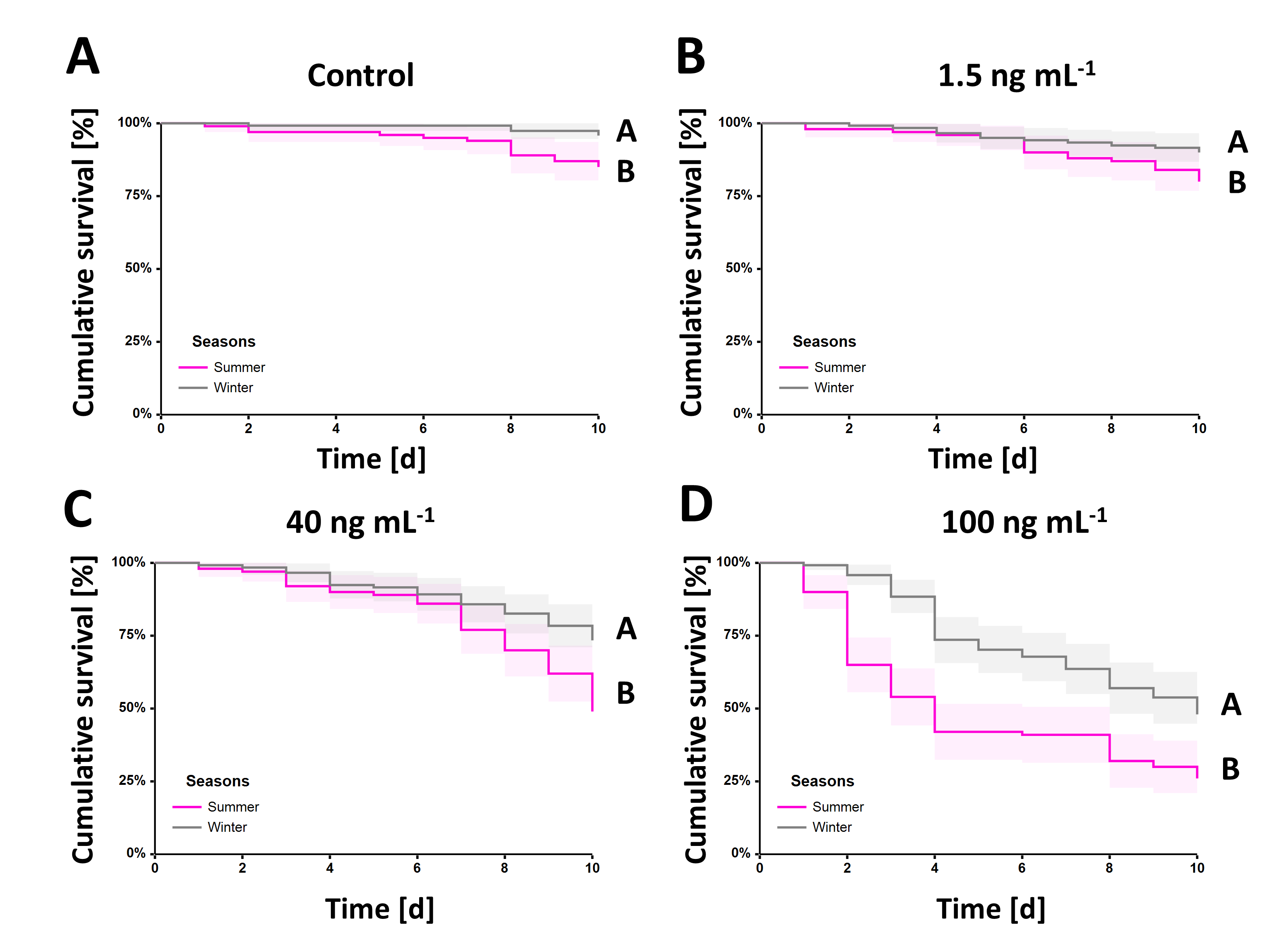
**

**Si Figure 2: Cumulative survival curves for summer and winter female (worker) bees, *Apis mellifera,* exposed to varying concentrations of thiamethoxam**. Kaplan-Meier cumulative survival curves show the survival over time for **(A)** 0 (i.e., control), **(B)** 1.5, **(C)** 40 and **(D)** 100 ng g ^-1^ thiamethoxam treatment groups. The solid grey (i.e., summer) and blue (i.e., winter) lines represent the survival rates of the honey bee workers across the two seasons. Significant differences (i.e., *p* < 0.05; Bonferroni corrected) are indicated the capitalized letters A and B.

**SI Table 1*:* Summary of statistical methods and results of the seasonal effects of the neonicotinoid thiamethoxam on honey bee workers, *Apis mellifera*.** The STATA16 model functions (e.g., *mestreg*) as well as the complete statistical output (e.g., *z* and *p-*values) are reported for each endpoint variable (e.g., daily consumption [g], daily exposure [ng] or survival [%]), along with the factors (i.e., fixed and random) included in the generalized linear/logistic mixed effects models for both seasons (i.e., summer **(A;** shaded yellow**)** and winter **(B;** shaded green**)**).

***
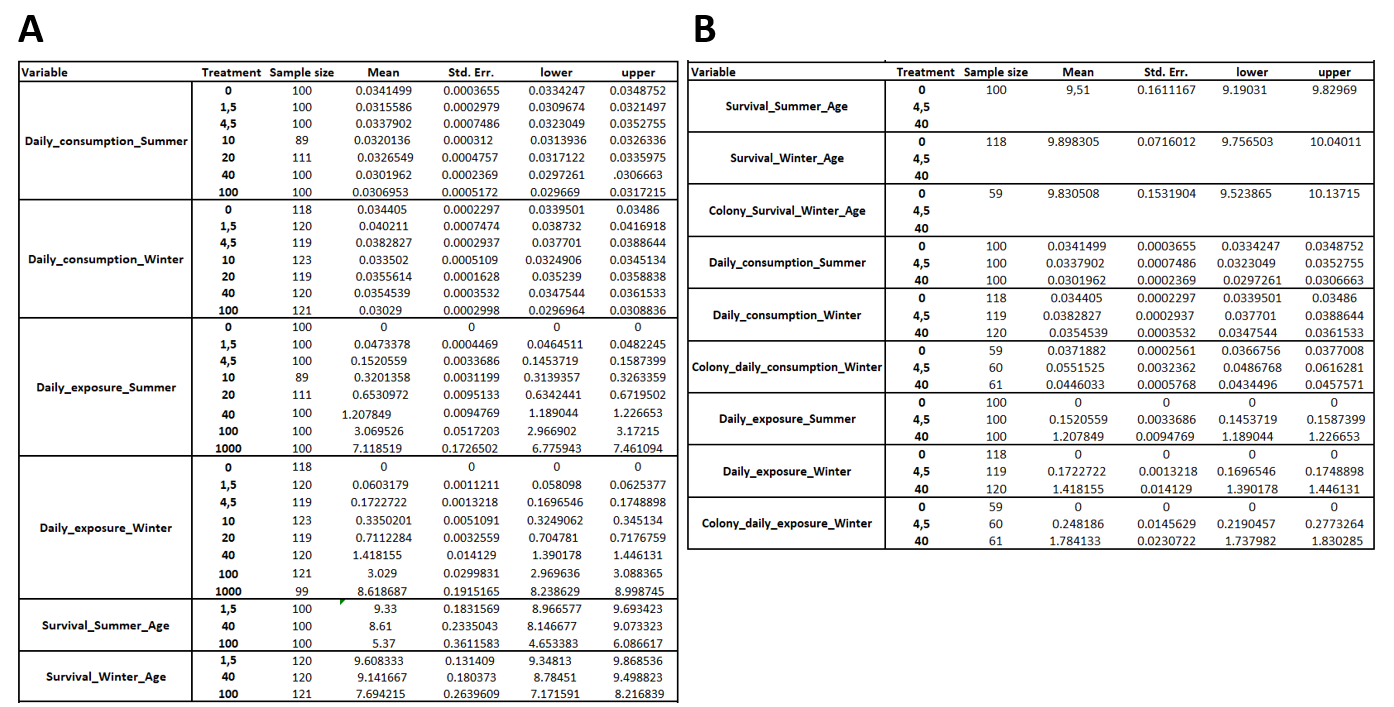
***

**SI Table 2: Summary of results of the seasonal effects of the neonicotinoid thiamethoxam on honey bee workers, *Apis mellifera*, for all outcome variables**. Sample size, mean, standard error and confidence interval are given for each **(A)** daily consumption, daily exposure and survival for summer and winter workers, as well as for **(B)** colony survival, daily consumption and daily exposure.
